# Fish proliferation and rare-earth deposition by topographically induced upwelling at the late Eocene cooling event

**DOI:** 10.1038/s41598-020-66835-8

**Published:** 2020-06-18

**Authors:** Junichiro Ohta, Kazutaka Yasukawa, Tatsuo Nozaki, Yutaro Takaya, Kazuhide Mimura, Koichiro Fujinaga, Kentaro Nakamura, Yoichi Usui, Jun-Ichi Kimura, Qing Chang, Yasuhiro Kato

**Affiliations:** 1 Frontier Research Center for Energy and Resources (FRCER), The University of Tokyo, 7-3-1 Hongo, Bunkyo-ku, Tokyo 113-8656 Japan; 2 Ocean Resources Research Center for Next Generation (ORCeNG), Chiba Institute of Technology, 2-17-1 Tsudanuma, Narashino, Chiba 275-0016 Japan; 3 Department of Systems Innovation, The University of Tokyo, 7-3-1 Hongo, Bunkyo-ku, Tokyo 113-8656 Japan; 4 Volcanoes and Earth’s Interior Research Center, Research Institute for Marine Geodynamics, Japan Agency for Marine-Earth Science and Technology (JAMSTEC), 2-15 Natsushima-cho, Yokosuka, Kanagawa 237-0061 Japan; 5 Submarine Resources Research Center, Research Institute for Marine Resources Utilization, Japan Agency for Marine-Earth Science and Technology (JAMSTEC), 2-15 Natsushima-cho, Yokosuka, Kanagawa 237-0061 Japan; 6 Department of Planetology, Kobe University, 1-1 Rokkodai-cho, Nada-ku, Kobe, Hyogo 657-8501 Japan; 7 Faculty of Science and Engineering, Waseda University, 3-4-1 Okubo, Shinjuku-ku, Tokyo 169-8555 Japan

**Keywords:** Palaeoceanography, Palaeoclimate, Geochemistry

## Abstract

The deep-sea clay that covers wide areas of the pelagic ocean bottom provides key information about open-ocean environments but lacks age-diagnostic calcareous or siliceous microfossils. The marine osmium isotope record has varied in response to environmental changes and can therefore be a useful stratigraphic marker. In this study, we used osmium isotope ratios to determine the depositional ages of pelagic clays extraordinarily rich in fish debris. Much fish debris was deposited in the western North and central South Pacific sites roughly 34.4 million years ago, concurrent with a late Eocene event, a temporal expansion of Antarctic ice preceding the Eocene–Oligocene climate transition. The enhanced northward flow of bottom water formed around Antarctica probably caused upwelling of deep-ocean nutrients at topographic highs and stimulated biological productivity that resulted in the proliferation of fish in pelagic realms. The abundant fish debris is now a highly concentrated source of industrially critical rare-earth elements.

## Introduction

Across the Eocene–Oligocene (E–O) boundary, ca. 33.9 million years ago (Ma), a large-volume ice sheet grew on Antarctica^1–3^. This event and subsequent development of the permanent polar ice sheets during the Cenozoic era occurred after a globally warm period in the early Eocene^1–3^. Several biological proxies extracted from deep-sea sediments have revealed a contemporaneous marine ecosystem shift and biological productivity changes in the Southern Ocean and equatorial Pacific Ocean^4,5^. However, deep-sea clays in oligotrophic, pelagic realms contain hardly any analogous proxies, such as calcareous or siliceous microfossils.

Microscopic fish skeletal debris are the only fossil remains well preserved in pelagic clay that otherwise lacks fossils. They are predominantly fish teeth and denticles—referred to as ichthyoliths—and bone fragments, all of which are composed of biogenic calcium phosphate^6^ (Supplementary Fig. S1a). Although they are usually a minor component of the biological proxies in sediments, fish skeletal debris have been effectively used to investigate the response of fish populations to Cenozoic environmental changes in the open ocean^7,8^. Moreover, the large amounts of fish debris in some pelagic clays from the Pacific^9^ and Indian^10^ Ocean have resulted in high bulk contents of rare-earth elements and yttrium (REY)^11^. Pelagic clay cores collected from the western North Pacific Gyre, for example, include a several-meters-thick layer that contains unusual amounts of fish debris^12,13^ and has a bulk REY content up to ~8,000 ppm^9,12^. These previous studies have demonstrated the spatiotemporal variability of fish debris concentrations in pelagic clay. This variability implies that abyssal clay has recorded biotic responses of fish to environmental changes even in the oligotrophic open ocean.

Determination of the depositional age of the pelagic clay containing the anomalous amounts of fish debris allowed us to unravel the causes of the anomalous accumulation of fish debris in the context of contemporaneous environmental changes and biotic responses. Here we determined the depositional ages of pelagic clay layers enriched in fish debris from the western North and central South Pacific Ocean by using a combination of the isotopic ratio of osmium ^187^Os/^188^Os in seawater^14^ and the stratigraphy of ichthyoliths^6^. The ^187^Os/^188^Os ratios in seawater have fluctuated in response to a balance between Os fluxes from continental (riverine), hydrothermal, and extraterrestrial sources^15^. Fe-oxyhydroxides in deep-sea sediments and ferromanganese (Fe-Mn) crusts record and preserve the ^187^Os/^188^Os ratios of seawater at the time of deposition. Therefore, comparison of the measured values of the samples with the marine ^187^Os/^188^Os curve^14,15^ enables determination of the depositional age of each sample^16^. Ichthyoliths can constrain the depositional ages of pelagic clays based on the stratigraphic ranges of ichthyolith species identified from the morphological features of ichthyoliths^6^.

## Results

### Lithologies of the studied sediment cores

**Figure 1:**
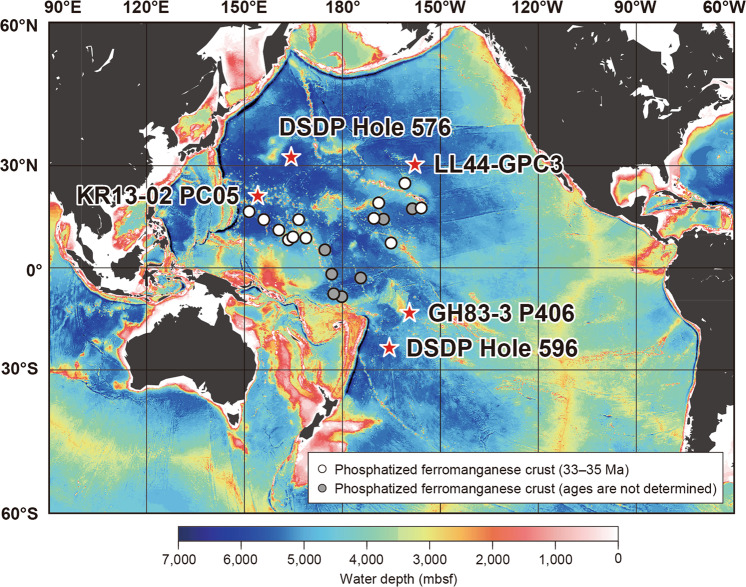
Locations of cores KR13-02 PC05, GH83-3 P406, DSDP Holes 596 and 576, and LL44-GPC3 with the bathymetry of the Pacific Ocean. Sampling sites of Fe-Mn crusts phosphatized during the interval 33–35 Ma^38^ and those whose phosphatization ages were not constrained^67^ are also shown. Bathymetric data are from ETOPO1^68^ (NOAA National Geophysical Data Center: 10.7289/V5C8276M; https://www.ngdc.noaa.gov/mgg/global/global.html). This map was created by using Generic Mapping Tools software, Version 4.5.18^69^ (https://www.soest.hawaii.edu/gmt/).

**Figure 2:**
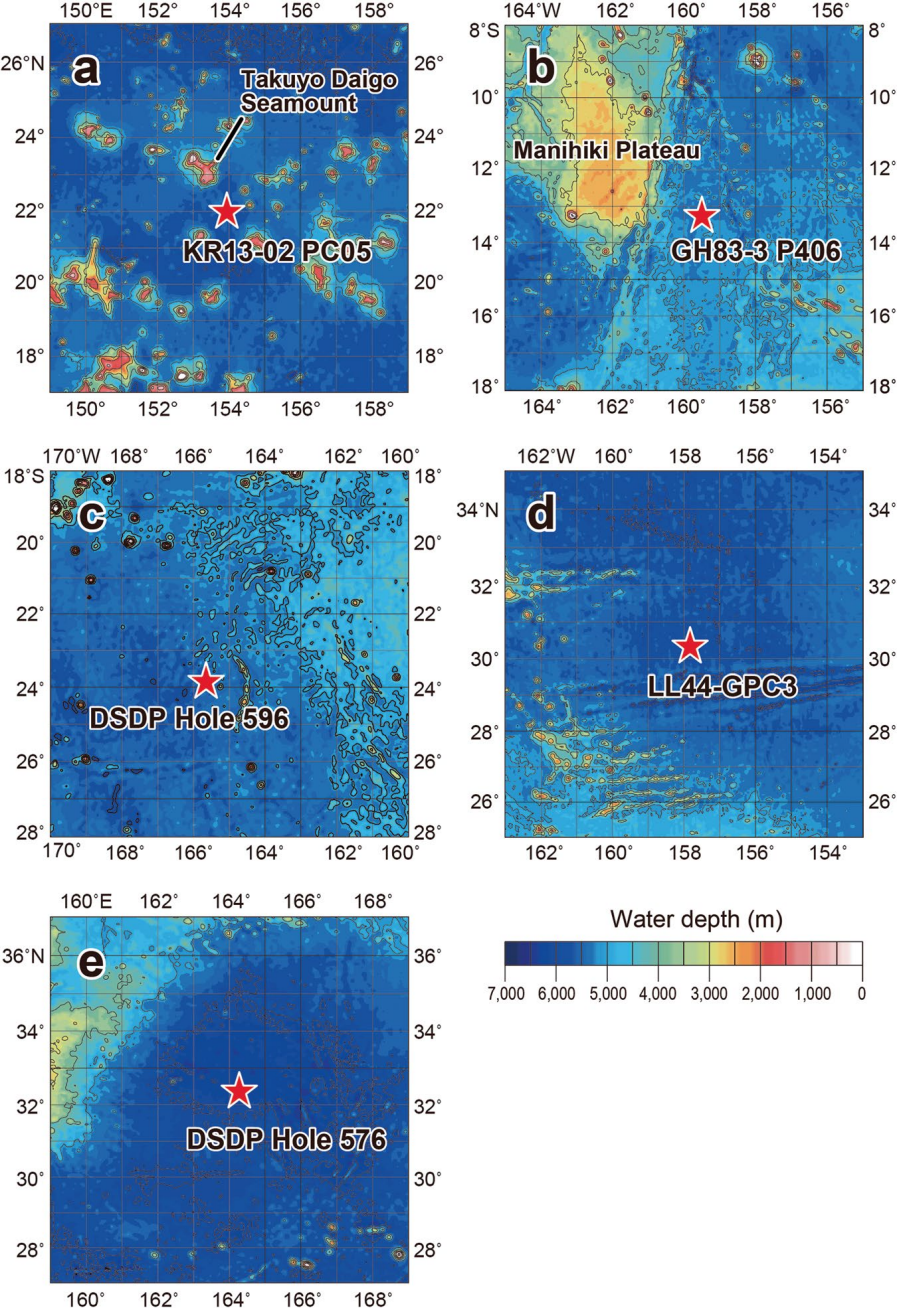
Detailed seafloor topographies around the study sites. (**a**) KR13-02 PC05, (**b**) GH83-3 P406, (**c**) DSDP Hole 596, (**d**) LL44-GPC3, and (**e**) DSDP Hole 576. Bathymetric data are from ETOPO1^68^ (NOAA National Geophysical Data Center: 10.7289/V5C8276M; https://www.ngdc.noaa.gov/mgg/global/global.html). This map was created with Generic Mapping Tools software, Version 4.5.18^69^ (https://www.soest.hawaii.edu/gmt/).

**Figure 3:**
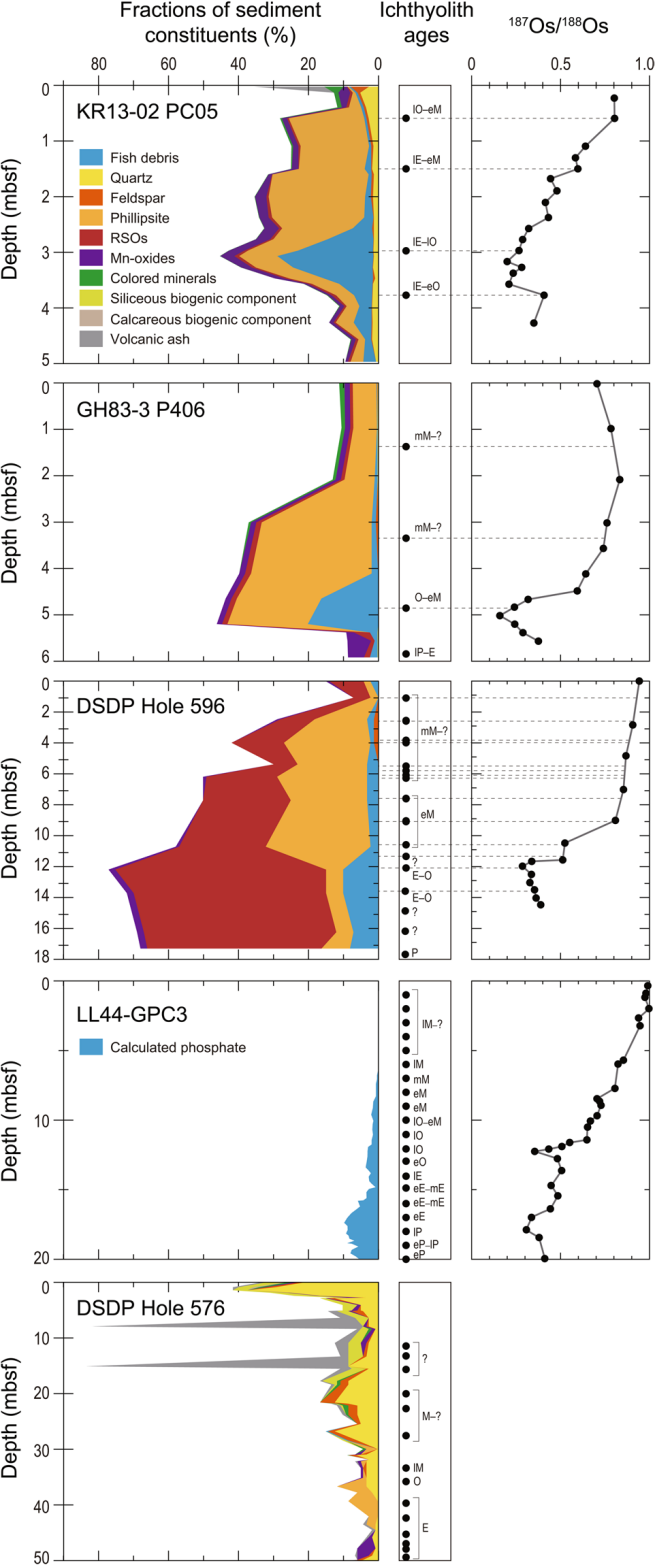
Downhole variations of fractions of sediment constituents, ichthyolith ages, and ^187^Os/^188^Os ratios in the cores used in this study. Fractions of sediment constituents of cores DSDP Hole 596^18^ and DSDP Hole 576^22^ and ichthyolith ages of cores GH83-3 P406^17^, DSDP Hole 596^42^, LL44-GPC3^19^, and DSDP Hole 576^20^ have been previously reported. The rest of the sediments consisted of clay-sized particles. The calculated phosphate content of core LL44-GPC3, an indicator of the amount of fish debris^21^, was used in this study instead of the reported low-resolution smear slide data^70^. The ^187^Os/^188^Os ratios of LL44-GPC3 has previously been reported^23^. The following abbreviations of epochs indicate ages based on ichthyolith stratigraphy: M = Miocene, O = Oligocene, E = Eocene, and P = Palaeocene. Abbreviations of epoch adjectives are as follows: e = early, m = middle, and l = late. Question mark means not constrained.

We targeted three pelagic clay core samples collected from the Pacific deep-sea floor for age determination (Fig. 1). Core KR13-02 PC05 was obtained from the deep-sea plain of the northern Pigafetta Basin in the western North Pacific during cruise KR13-02 conducted by the Japan Agency for Marine-Earth Science and Technology (JAMSTEC)^12^ (Figs. 1 and 2a). Core GH83-3 P406 was obtained from the Penrhyn Basin in the central South Pacific during cruise GH83-3 conducted by the Geological Survey of Japan (GSJ)^17^ (Figs. 1 and 2b). Core samples from Deep Sea Drilling Project (DSDP) Hole 596 were obtained from the Southwest Pacific Basin during DSDP Leg 91^18^ (Figs. 1 and 2c). Lithologies of these cores^12,17,18^ are typical pelagic clay composed mainly of clay-sized siliciclastic particles with significant amounts of zeolite (phillipsite) (Fig. 3). The fish debris fraction of core KR13-02 PC05 was less than 5% at depths more than 3.8 meters below the seafloor (mbsf) and peaked at nearly 30% about 3.1 mbsf (Fig. 3, and Supplementary Table S1 and Fig. S2). It then decreased to less than 3% above 2.4 mbsf. The fish debris fraction of core GH83-3 P406, which was less than 2% below 5.4 mbsf, peaked at approximately 20% at 5.2 mbsf and then decreased to less than 1% above 3.0 mbsf (Fig. 3, and Supplementary Table S1 and Fig. S3). In DSDP Hole 596, the fish debris fraction reached 7–10% at depths below 12.2 mbsf and was less than 3% above 10.7 mbsf^18^ (Fig. 3). We compared these three cores with two other pelagic clay cores, LL44-GPC3 and DSDP Hole 576 (Figs. 1, and 2d,e), the depositional ages of which had already been constrained by ichthyolith stratigraphy^19,20^. The calculated fraction of phosphate in core LL44-GPC3, an indicator of fish debris accumulation^21^, was 7–10% at depths below 15 mbsf and less than 5% above 15 mbsf^21^ (Fig. 3). In DSDP Hole 576, the fraction of fish debris was only 1% from 26.8 to 28.5 mbsf^22^ (Fig. 3).

### Marine ^187^Os/^188^Os records in the study cores

The ^187^Os/^188^Os records in cores KR13-02 PC05, GH83-3 P406, and DSDP Hole 596 showed common features (Fig. 3 and Supplementary Table S2). The ^187^Os/^188^Os ratios of the cores showed radiogenic minima of <0.3 at 3.2 mbsf in core KR13-02 PC05, at 5.0 mbsf in core GH83-3 P406, and at 11.0 mbsf in core DSDP Hole 596. Above the horizons associated with these radiogenic minima, the ratios gradually increased with decreasing depth. Near the top of the cores, the ratios increased to radiogenic values of 0.7–1.0. The depth profiles and the ranges of our new ^187^Os/^188^Os data were consistent with those of the marine ^187^Os/^188^Os curve^14,15^ in the time interval from the late Eocene to the present (Fig. 3 and Supplementary Fig. S4). The previously reported Os isotope compositional data of LL44-GPC3^23^ above 14.0 mbsf showed a trend similar to that of the three cores described above (Fig. 3). The ^187^Os/^188^Os ratio showed a radiogenic minimum at 12.2 mbsf and then increased with decreasing depth.

### Age assignments and calculated fish debris accumulation rates

**Figure 4:**
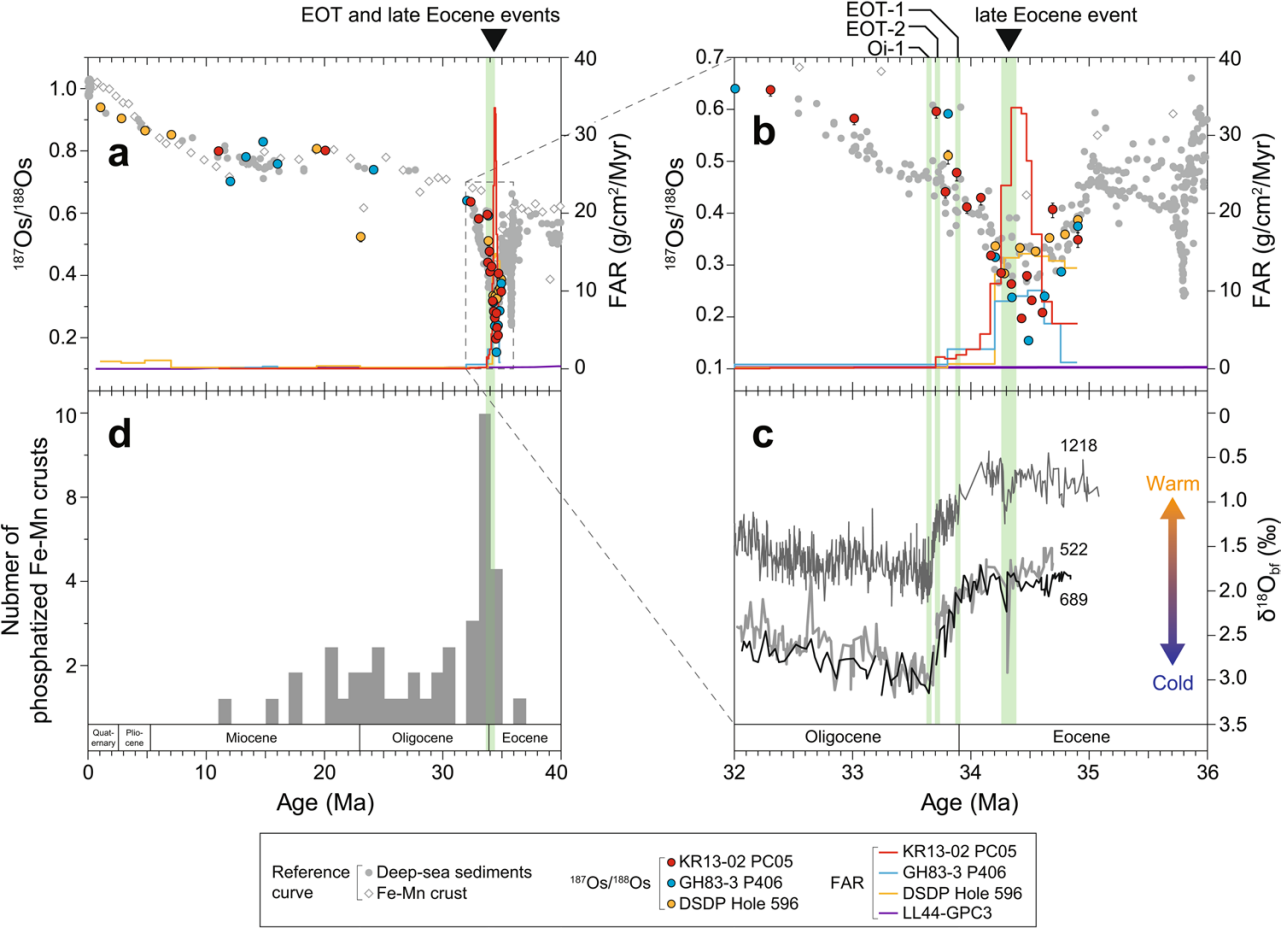
The age-assigned ^187^Os/^188^Os records in the study cores with fish debris accumulation rates (FARs), age distributions of phosphatized Fe-Mn crusts, and *δ*^18^O_bf_ records. (**a**) ^187^Os/^188^Os records in cores KR13-02 PC05, GH83-3 P406, and DSDP Hole 596, with the FARs of these cores and the calculated phosphate accumulation rate of LL44-GPC3^21^, from 40 Ma to the present. The ages of the samples were assigned based on our age determination procedure. (**b**) Enlarged view of (a) from 36 to 32 Ma. The green shaded lines in (**a**,**b**) indicate the EOT and the late Eocene events. Error bars on ^187^Os/^188^Os data points indicate 2 S.D. plus the differences between measured values and ^187^Re decay–corrected initial values (see Methods). Note that error bars of almost all samples are smaller than symbols. (**c**) *δ*^18^O_bf_ obtained from DSDP Sites 522^71^ and 689^72^, and ODP Site 1218^5^. Ages of these cores were calibrated by adjusting biostratigraphic and magnetostratigraphic age benchmarks based on the chronology of the Geologic Time Scale 2012^62^. (**d**) Histogram of the ages of phosphatized Fe-Mn crust samples from the Pacific Ocean^38^.

The remarkably low ^187^Os/^188^Os minima in the cores were all attributable to the ^187^Os/^188^Os excursion across the E–O boundary (Fig. 4a,b, and Supplementary Fig. S5). Our age determination procedure based on the combination of Os isotope ratios and ichthyolith stratigraphy is fully described in Methods. The fact that the combined stratigraphy successfully determined the depositional ages of all the sampling points allowed us to reconstruct records of accumulation rates of fish debris (including teeth, denticles, and bone fragments) at each site. These records demonstrated significant changes in the fish debris accumulation rates (FAR) around the E–O boundary at the study sites (Fig. 4a,b). From 34.7 to 34.4 Ma, the FAR at the site of core KR13-02 PC05 increased abruptly to about six times the FAR before 34.7 Ma and peaked between 34.4 and 34.3 Ma. The FAR then decreased abruptly to a value lower than before 34.7 Ma. The FAR record of core GH83-3 P406 showed a similar pattern to that of core KR13-02 PC05. From 34.8 to 34.6 Ma, the FAR increased abruptly to about 12 times the value before 34.8 Ma and peaked between 34.3 and 34.2 Ma. The FAR subsequently decreased abruptly at 34.2 Ma, and after 33.8 Ma it decreased to a value similar to that before 34.8 Ma. In contrast, at 34.9 Ma the FAR was higher at the site of DSDP Hole 596 than at the other study sites but did not show any prominent increase until 34.3 Ma. In our record, the FAR abruptly decreased at 34.2 Ma to a rate much lower than the FARs of the other study cores. This abrupt decrease probably corresponded to the hiatus at 11.7 mbsf that was suggested from the bulk chemical composition^24^ (Supplementary Fig. S5c,f). The record at the site of DSDP Hole 596 indicated that the FAR suddenly dropped at 34.2 Ma, and that there was no increase before that drop. A previously reported record of the accumulation rate of fish teeth and denticles at the site of DSDP Hole 596^8^ and our FAR record at the same site agreed with each other in that the rates were nearly constant and without any prominent increases in the time period from the late Eocene to the early Oligocene. This comparison indicates that our FAR record is consistent with the long-term trend of fish debris accumulation, although the previous study counted only teeth and denticles^8^, whereas our FAR included all phosphatic fish debris (denticles, teeth, and bones). Although the age models at the site of LL44-GPC3 were based on ichthyolith stratigraphy and a constant cobalt flux model that differed from our age model, the mass accumulation rate of phosphate showed no obvious increase around the E–O boundary. In core DSDP Hole 576, a detectable amount (>1%) of fish debris was not observed in the Eocene to Oligocene sequence, based on a smear slide analysis^22^ (Fig. 3). In summary, prominent and transient increases of fish debris accumulation occurred at the sites of KR13-02 PC05 and GH83-3 P406 around 34.5 Ma in the very late Eocene but did not occur at the sites of DSDP Hole 596, LL44-GPC3, and DSDP Hole 576.

## Discussion

From the middle to late Eocene, the opening of the Southern Ocean Gateways—the Drake Passage and Tasman Rise—led to the development of a current around Antarctica shallower than the present Antarctic Circumpolar Current (ACC), the so-called proto-ACC^2,25^. The possibility that a deep water mass began to form around Antarctica in the late Eocene (ca. 36.5 Ma) and subsequently spread into the North Pacific is supported by the gradual increase of the oxygen isotope ratios of benthic foraminifera (*δ*^18^O_bf_) over a period of about 2.5 million years^25^.

Subsequently, a critical environmental change occurred around 34 Ma. That change is given special recognition as the E–O climate transition (EOT) and is marked by the onset of a permanent ice sheet on Antarctica, changes of ocean circulation, and a climatic regime shift^1,2^. These changes have been attributed to a decrease of atmospheric CO_2_ concentrations^26,27^, as well as to the opening of the Southern Ocean gateways, which led to thermal isolation of Antarctica^2,28^. In contrast to the secular changes of ocean circulation due to the opening of the gateway^25^, the ice-sheet growth is recognized to have occurred in several steps that involved short-term increases of the ice volume on Antarctica^5,29,30^. The Pacific and Atlantic *δ*^18^O_bf_ records have revealed two minor positive shifts called EOT-1 and EOT-2^5,29^ (Fig. 4c). These shifts were followed by a major positive shift referred to as Oi-1 that corresponds to the onset of the permanent Antarctic ice-sheet^1,2,5,29,30^ (Fig. 4c). About 1 million years before Oi-1, some *δ*^18^O_bf_ records display a transient increase by ~0.5‰, which is called the late Eocene event^5,29^ (Fig. 4c). During that time, ice-volume, seawater temperature, and sea-level changes, based on *δ*^18^O_bf_, the Mg/Ca ratios of foraminiferal shells, and geological records, respectively, collectively indicate expansion of the Antarctic ice volume without changes of tropical sea surface temperature^29^. Previously reported records of biogenic silica and carbonate accumulation rates in the Southern Ocean have suggested that productivity increased in response to the Oi-1 glaciation^4^. In the eastern Equatorial Pacific Ocean, the record of the benthic foraminifera accumulation rate, a proxy for palaeoproductivity, shows a transient increase at the time of the Oi-1 glaciation^5^. These simultaneous increases of productivity have been attributed to intensification of oceanic and atmospheric circulation that drove divergence and upwelling of nutrient-rich thermocline waters in these regions^4,5^.

Because fish are consumers or predators in an ecosystem, the fish population likely reflects surface ocean productivity^31^. Therefore, the significant increase of FAR at the sites of KR13-02 PC05 and GH83-3 P406 may have resulted from greater biological productivity and a larger population of fish at these sites associated with ocean circulation changes toward the end of the Eocene. However, the significantly shorter duration of the FAR increases compared to the time required for reorganization of ocean circulation in response to the gateway opening indicates that the FAR increases were instead related to a short-term event. In addition, the peak of productivity at these sites preceded the major glaciation event of Oi-1 by about 1 Myr (Fig. 4b,c). Moreover, there were no significant increases of productivity at the contemporary sites of DSDP Hole 596, LL44-GPC3, and DSDP Hole 576, although they, as well as the sites of KR13-02 PC05 and GH83-3 P406, are located in pelagic areas. It is probable that the higher FAR at 34.9 Ma at the site of DSDP Hole 596 in the South Pacific versus the other sites (Fig. 4b) is a reflection of high biological productivity in the South Pacific during the late Eocene^32,33^. The differences in the patterns between our records and those from the Southern Ocean^4^ and eastern Equatorial Pacific Ocean^5^—that is, the timing and spatial pattern of the productivity changes—suggest a fundamental difference in the mechanisms that caused the productivity increases. The productivity increases at the sites of KR13-02 PC05 and GH83-3 P406 coincided with the late Eocene event at ~34.4 Ma (Fig. 4b,c), and their durations were more likely equivalent to the time associated with the *δ*^18^O_bf_ increase of the late Eocene event than to the duration of the secular *δ*^18^O_bf_ change caused by the gateway opening^25^. The implication is that there was a close relationship between the productivity increases at the two sites and the late Eocene event.

A coupled ocean-atmosphere model that tested the role of the Antarctic ice sheet on changes of ocean circulation during the EOT has demonstrated that growth of the Antarctic ice sheet could have invigorated Antarctic bottom water formation as a result of the high southern latitude cooling^34^. Therefore, the expansion of the ice sheet during the late Eocene event, although smaller than the expansion at the Oi-1, would have stimulated northward flow of bottom water by cooling Antarctica. Because the circulation of the Eocene ocean was relatively sluggish prior to the initial formation of the Antarctic ice sheet at the late Eocene event, nutrients could have accumulated in the deep ocean^35^. The intensified northward flow would have resulted in the first stirring of nutrient-rich deep ocean water. This stirring would have led to an upwelling of nutrients to the surface ocean in regions with topographic barriers that were steep and large enough to allow upwelling^35^ (Supplementary Fig. S6). In the modern ocean, enhanced primary productivity around seamounts is recognized to be a result of upwelling generated by seamount–current interactions^31,36^. Though seamount–current interactions are complicated oceanographic processes, these currently observed increases of productivity support the hypothesized nutrient upwelling during the late Eocene event. The seamount chains in the present western North Pacific Ocean around KR13-02 PC05 and the Manihiki Plateau near site GH83-3 P406 can likewise enhance upwelling of bottom water (Fig. 2a,b). This supply of nutrients for the first time in oligotrophic pelagic realms may have been sufficient to allow pelagic organisms to flourish. The osmium isotope records (Fig. 4b) suggest that this enhancement of production continued for about a hundred thousand years, at the end of which time the nutrients stored in the deep water of these regions had been consumed and dispersed, and the enhancement of production ceased. This mechanism can explain the differences between the records of palaeoproductivity at the other sites. Because the sites of DSDP Hole 596, LL44-GPC3, and DSDP Hole 576 are located on a vast deep-sea plain without any topographic rises near the sites (Figs. 1 and 2c–e), there would have been no upwelling of nutrient-rich, northward-flowing water.

Other geological observations support this postulated mechanism of enhanced production. The bimodal grain size distribution of the fish debris-rich layer in core KR13-02 PC05, with peaks at ~4 µm and 60–80 µm, indicates a winnowing of the fine silt-sized fraction^13,37^ (Supplementary Fig. S7). In addition, the grain sizes of fish debris and phillipsite (volcanic in origin) were coarser in the layer rich in fish debris than in the other parts of the sediment column^13^ (Supplementary Figs. S2 and S3). The similar grain size patterns of the fish debris and phillipsite, which have different sources (Supplementary Fig. S1), suggests hydraulic fractionation caused by enhanced flow of bottom water^13,37^. Moreover, active upwelling of phosphate-rich bottom water over the seamount could have promoted phosphatization of Fe-Mn crusts on the central Pacific seamounts from the very late Eocene to the earliest Oligocene (Figs. 1 and 4d). The result would have been a drastic redistribution of phosphorus in the ocean^35,38^. The geochemical and geological lines of evidence suggest that significant amounts of fish debris could have been deposited on the deep seafloor in the pelagic realm where there were steep and large topographic rises in the late Eocene and where there was an enhanced northward flow of bottom water formed as a result of the ephemeral expansion of the Antarctic ice sheet superimposed on a secular reorganization of ocean circulation.

The synchronous proliferation of fish during the late Eocene event in the western North and central South Pacific sites provides new insights into the distributions of rare-earth elements in the Pacific Ocean sediments. Fossilization of fish debris in pelagic clay led to highly concentrated REY—up to ~20,000 ppm of total REY^9^. Pelagic clay rich in fish debris can therefore be a promising new source of REY for industry. The distribution of such REY-rich mud^9^ is very heterogeneous, even in a relatively small area. Bulk chemical composition analyses of the sediment cores in an area of 2,500 km^2^ in the southern plain at the foot of the Takuyo Daigo Seamount^9,12,39^ (Supplementary Fig. S8), including core KR13-02 PC05, have indicated that the REY content is very inhomogeneous. A lithological study of the sediment cores (including core P406) collected by the GH83-3 cruise in an area of roughly 100 km × 100 km has also revealed a large variation in their lithologies^17^. Although these heterogeneities make it difficult to precisely estimate the amount of the REY-rich mud resource, a previous study has shown that REY-rich mud containing large amounts of fish debris to a depth of 10 mbsf in an area of 2,500 km^2^ around the site of KR13-02 PC05 (Supplementary Fig. S8) can provide as much REY as several hundred years of consumption in the modern world^9^.

Pelagic clays rich in fish debris have been found in only two areas in this study, KR13-02 PC05 and GH83-3 P406 area, but the mechanism of blooming that we proposed in this study allows us to constrain potential areas where similar clays may be found in the Pacific Ocean. On the assumption that such clays could form at the foot of steep topographic rises from ocean basins at depths greater than ~5,000 m (i.e., well below the carbonate compensation depth) and had a relative elevation of several thousands of meters (i.e., sufficiently high to induce an upwelling of bottom water to the surface ocean), the potential area could cover a wide region through the Pigafetta Basin in the western North Pacific, Mid Pacific Mountains, and Penrhyn Basin in the central South Pacific (Supplementary Fig. S6). The actual targets are expected to be relatively small areas located at the foot of steep slopes in the potential area. If this assessment is correct, the clay might be a huge storehouse of elements associated with fish debris such as REY and phosphorous.

We conclude that marine organisms in the pelagic realm of the Pacific Ocean had flourished in response to the fluctuation of oceanic circulation caused by Antarctic cooling during the late Eocene event that preceded the EOT. The intensified northward flow of bottom water formed around Antarctica resulted in the first stirring of nutrient-rich deep ocean water, which led to an upwelling of nutrients to the surface ocean in regions with topographic highs. The global change of the Cenozoic climate also facilitated the incorporation of phosphorus-favoured elements such as REY into pelagic clay.

## Methods

### Smear slide analysis

The fractions of sediment constituents in some horizons of cores KR13-02 PC05 and GH83-3 P406 were determined by smear slide analyses following the protocols of the Ocean Drilling Project (ODP)^40^ (Supplementary Table S1). We identified clay-sized particles, quartz, feldspar, phillipsite, fish debris, red-brown to yellow-brown semi-opaque oxides (RSOs)^41^, micro-manganese oxides (Mn-oxides), coloured minerals, and volcanic ash. Microphotographs of the representative horizons are shown in Supplementary Figs. S2 and S3.

### Ichthyolith stratigraphy

The ages of cores GH83-3P406 and DSDP Hole 596 had been previously constrained by ichthyolith stratigraphy^17,42^. For core KR13-02 PC05, approximately 3 g of wet sediment sample was sieved through 62-µm–opening mesh with Milli-Q water. The obtained coarse portions were dried at 40 °C. The amount of the coarse portion of KR13-02 PC05, section 4, 72–74 cm, was reduced by random sampling. All teeth-like fragments contained in each coarse portion were handpicked and enclosed on glass slides using ultraviolet curing resin. Ichthyoliths were identified under a polarizing microscope based on previously published databases of systematic ichthyolith taxonomy^20,43–46^. The ichthyolith ages were determined as ranges of ages within which the taxa identified in each sample occurred together. The ichthyolith taxa that occurred in core KR13-02 PC05 are listed in Supplementary Table S3. Microphotographs of representative specimens of ichthyolith species identified in core KR13-02 PC05 are shown in Supplementary Fig. S9.

### Os and Re isotope analyses

Our analytical procedures for Os and Re isotopes have been fully described elsewhere^47,48^. All data obtained from the analyses are listed in Supplementary Table S2. Approximately 1 g of each dried and powdered bulk sediment sample was weighed and spiked with ^185^Re and ^190^Os. The samples were then digested in 4 mL of inverse aqua regia in sealed Carius tubes at 220 °C for 24 h to extract the seawater-derived Os from the pelagic sediment^49–51^. Solutions were separated from residues and diluted with Milli-Q water. The Os isotope ratios were measured with a Thermo Scientific Neptune multiple collector inductively coupled plasma mass spectrometer (MC-ICP-MS) at the Department of Solid Earth Geochemistry, JAMSTEC, combined with sparging sample introduction. Measurements of samples were performed by multiple Faraday collectors (FC), and those for procedural blank samples were performed with multiple-ion counters. Os concentrations were determined by isotope dilution and corrected for Os in the blank samples, the average of which was 0.36 ± 0.35 pg with a ^187^Os/^188^Os ratio of 0.140 ± 0.051 (n = 5; average ± 1 S.D.).

After the Os measurements, the solutions were heated to remove remaining Os. Re in the sample solutions was purified by two consecutive anion exchange chromatography steps. Measurements of Re for samples and blank samples were performed with an Agilent 7500ce ICP quadrupole mass spectrometer at the Super-cutting-edge Grand and Advanced Research (SUGAR) Program, Institute for Extra-cutting-edge Science and Technology Avant-garde Resarch (X-star), JAMSTEC. Concentrations of Re were determined by isotope dilution and corrected for Re in the blank samples, the average of which was 4.28 ± 1.93 pg (n = 5; average ± 1 S.D.). Concentrations of Re were used to correct measured ^187^Os/^188^Os data and to calculate initial ^187^Os/^188^Os values, as described below.

In addition to the samples from the study cores, we measured several uppermost sediment samples (0.00–0.02 mbsf) collected near core KR13-02 PC05 on cruises KR13-02 and MR14-E02 (Supplementary Table S3) to confirm that our Os isotopic data represented marine ^187^Os/^188^Os values. The ^187^Os/^188^Os ratios of these samples ranged from 0.954 ± 0.011 to 0.997 ± 0.012 (Supplementary Table S3). These values are generally consistent with the modern seawater value in the central Pacific (1.044 ± 0.03640^52^). This result confirmed that the ^187^Os/^188^Os data obtained with our analytical procedures reflected seawater values. The ^187^Os/^188^Os ratio of the sample near the top of core KR13-02 PC05 (section 1, 2–4 cm) showed a very atypical radiogenic value (0.465) and much lower Os concentration (63.49 pg/g) than the corresponding values in the uppermost sediment samples (0.954–0.997 and 109.13–132.21 pg/g, respectively). We examined a smear slide of this sample under a polarizing microscope and confirmed that this sample contained larger amounts (up to ~50%) of volcanic components such as ash and coloured minerals compared to the other samples. This result indicated that the ^187^Os/^188^Os ratio in this sample was probably disturbed by non-radiogenic Os in the volcanic components. Thus, this sample was excluded from the age-assignment procedure described below.

### Marine ^187^Os/^188^Os curve since 40 Ma

To determine the age of our samples, we compiled a marine ^187^Os/^188^Os curve from 40 Ma to the present (Supplementary Fig. S4) based on several published ^187^Os/^188^Os data obtained from deep-sea sediment cores^53–61^. The ages of the cores used in these previous studies were tightly constrained by biostratigraphy and magnetostratigraphy. In addition, we calibrated their ages by adjusting the biostratigraphic and magnetostratigraphic age benchmarks based on the chronology of the Geologic Time Scale 2012^62^. Thus, this marine ^187^Os/^188^Os curve represented the most precise age–^187^Os/^188^Os relationship in the ocean. Most previous studies have reported high-resolution marine ^187^Os/^188^Os data from ~37 to ~32 Ma and from ~15 Ma to the present, but the temporal resolution of the data in other periods has been relatively low. To complement the reference curve, we employed a long-term record of marine ^187^Os/^188^Os ratios obtained from a Fe-Mn crust in the Pacific^63^ refined by the thallium isotope record in the crust^16^ (Supplementary Fig. S1). Although there are slight discrepancies in the details of these records, they show generally consistent trends throughout the time interval from 40 Ma to the present.

### Procedure of age assignment based on the marine ^187^Os/^188^Os record

In previous studies that determined ages using an approach similar to this study^16,64^, several age benchmarks (e.g., 0 Ma for the present marine ^187^Os/^188^Os ratio and a prominent negative excursion around the E–O boundary) were identified, and constant growth rates were assumed between these benchmarks. However, pelagic clays are known to often include sedimentation hiatuses or periods of extremely slow sedimentation^65^. Thus, the above approach may have resulted in incorrect age assignments for pelagic clays. To avoid this problem, we identified the age of an individual sample by comparing its ^187^Os/^188^Os ratio with the age–^187^Os/^188^Os relationship of the marine ^187^Os/^188^Os curve. The final age assignments were obtained when the best fits were achieved after slight adjustments of fitting not to exceed typical sedimentation rates of pelagic clay (<5 m/Myr)^66^. Here, we describe how we determined the ages of our samples. To simplify the description, we assigned a sample number (s.n.) to each sample used for the age determination (Supplementary Table S2 and Fig. S5).

In core KR13-02 PC05 (Supplementary Fig. S5a,d), s.n. 1 (^187^Os/^188^Os = 0.801) corresponded to 11 Ma on the marine ^187^Os/^188^Os curve. Then, s.n. 2 (^187^Os/^188^Os = 0.801), for which the ichthyolith age represented late Oligocene to early Miocene, should correspond to 20 Ma in the Fe-Mn crust record. The rest of the samples (s.n. 3 to 18; ^187^Os/^188^Os = ~0.2–0.6) were characterized by a negative shift of the ^187^Os/^188^Os and thus could be fitted to two prominent negative excursions at 35.8 Ma and across the E–O boundary. Use of a typical sedimentation rate of pelagic clay (<5 m/Myr)^66^ gave a reasonable fit of the series of ^187^Os/^188^Os values to the broader negative excursion across the E–O boundary. For the best fit, s.n. 3 to 5 were assigned to between 32.3 and 33.7 Ma, and s.n. 6 to 18 were assigned to between 33.8 and 34.9 Ma. These age assignments for core KR13-02 PC05 are consistent with the age constraints based on ichthyolith stratigraphy (Supplementary Table S3).

For core GH83-3 P406 (Supplementary Fig. S5b,e), s.n. 1 to 4 (^187^Os/^188^Os = ~0.7–0.8) could be assigned to between 12 and 16 Ma, where the Fe-Mn crust record shows a small fluctuation. The precise age of s.n. 5 (^187^Os/^188^Os = 0.740) could not be specified because it corresponded to a long-term plateau in the marine ^187^Os/^188^Os curve. The age of this sample was therefore tentatively assigned by assuming a constant sedimentation rate between s.n. 4 and 6. The rest of the samples (s.n. 6 to 13; ^187^Os/^188^Os = ~0.6–0.2), which were characterized by a negative shift of ^187^Os/^188^Os ratios, could be assigned to the relatively broad negative excursion across the E–O boundary for the same reason as in core KR13-02 PC05. For the best fit, s.n. 6 and 7 were assigned to 32.0 and 32.8 Ma, and s.n. 8 to 13 were assigned to 34.2 and 34.9 Ma, respectively. These age assignments for core GH83-3 P406 are consistent with the age constraints based on ichthyolith stratigraphy^17^, with the exception of s.n. 8 and 9, where the ichthyolith age represented the Oligocene to early Miocene. Considering that the resolution of the ichthyolith age was approximately epoch level^6^, the age assignments of these samples were very close to the E–O boundary and not necessarily inconsistent with the ichthyolith age.

For core DSDP Hole 596 (Supplementary Fig. S5c,f), s.n. 1 to 4 (^187^Os/^188^Os = ~0.9) were assigned to ages between 1 to 7 Ma. The s.n. 5 (^187^Os/^188^Os = 0.808), where the ichthyolith age represented early Miocene^42^, could correspond to ~19–21 Ma of the Fe-Mn crust record. The s.n. 6 (^187^Os/^188^Os = 0.524), where the ichthyolith age represented early Miocene^42^, could be assigned to ~23 Ma, where the Fe-Mn crust record showed a transient decrease. The rest of the samples (s.n. 7 to 14; ^187^Os/^188^Os = ~0.3–0.4) were associated with a negative shift of ^187^Os/^188^Os ratio and could be assigned to the broader negative excursions across the E–O boundary for the same reason as in cores KR13-02 PC05 and GH83-3 P406. For the best fit, s.n. 7 was assigned to 33.8 Ma, and s.n. 8 to 14 were assigned to between 34.2 and 34.9 Ma. These age assignments for core DSDP Hole 596 were consistent with the age constraints based on ichthyolith stratigraphy^42^. After the age assignments, we checked differences between measured ^187^Os/^188^Os values used in the age assignment procedures and the initial ^187^Os/^188^Os values calculated by subtracting the radiogenic ^187^Os generated from internal ^187^Re decay after deposition (Supplementary Table S2). The fact that the differences between the corrected initial ^187^Os/^188^Os values and uncorrected measured values (0.02–1.62%) were less than or almost equivalent to the analytical errors (0.30–3.61%; 2 S.D.) indicates that ^187^Re decay was negligible for the age assignment procedure.

### Dry bulk density, sedimentation rate, and mass accumulation rate

The dry bulk density (DBD) of core KR13-02 PC05 was measured at several depths. The DBD of core DSDP Hole 596 had previously been reported^18^. The DBD of core GH83-3 P406 has not yet been reported and was assumed to be constant and equal to 0.40 g/cm^3^, which is comparable to the average DBD of core DSDP Hole 596. We calculated the DBDs of cores KR13-02 PC05 and DSDP Hole 596 by linear interpolation of the measured DBDs; the Os isotope ratios of the same cores were measured (Supplementary Table S2). After determining the ages of cores KR13-02 PC05, GH83-3 P406, and DSDP Hole 596, we calculated the sedimentation rate (SR) in meters per million years (m/Myr) between sample *i* and *i* + 1 (*SR*_*i,i*+1_) and the FAR in g/cm^2^/Myr between sample *i* and *i* + 1 (*FAR*_*i,i*+1_) with the following equations:

$$\begin{array}{ccl}S{R}_{i,i+1} & = & \frac{{d}_{i+1}-{d}_{i}}{{t}_{i+1}-{t}_{i}}\\ FA{R}_{i,i+1} & = & S{R}_{i,i+1}\times DB{D}_{i,i+1}\times f{F}_{i,i+1}\end{array}$$

where *d*_*i*_ is the depth of sample *i* from the seafloor in meters, *t*_*i*_ is the age in millions of years of sample *i* determined in this study, *DBD*_*i,i*+1_ is the average DBD between sample *i* and *i*+1, and *fF*_*i,i*+1_ is the average fraction of fish debris between sample *i* and *i*+1.

## Supplementary information

Supplementary information.

## Data Availability

All data generated or analysed during this study are included in this published article and its Supplementary Information Files.
