# Author Correction: Fish proliferation and rare-earth deposition by topographically induced upwelling at the late Eocene cooling event

**DOI:** 10.1038/s41598-020-69875-2

**Published:** 2020-07-31

**Authors:** Junichiro Ohta, Kazutaka Yasukawa, Tatsuo Nozaki, Yutaro Takaya, Kazuhide Mimura, Koichiro Fujinaga, Kentaro Nakamura, Yoichi Usui, Jun-Ichi Kimura, Qing Chang, Yasuhiro Kato

**Affiliations:** 1 Frontier Research Center for Energy and Resources (FRCER), The University of Tokyo, 7-3-1 Hongo, Bunkyo-ku, Tokyo, 113-8656 Japan; 2 Ocean Resources Research Center for Next Generation (ORCeNG), Chiba Institute of Technology, 2-17-1 Tsudanuma, Narashino, Chiba 275-0016 Japan; 3 Department of Systems Innovation, The University of Tokyo, 7-3-1 Hongo, Bunkyo-ku, Tokyo, 113-8656 Japan; 4 Volcanoes and Earth’s Interior Research Center, Research Institute for Marine Geodynamics, Japan Agency for Marine-Earth Science and Technology (JAMSTEC), 2-15 Natsushima-cho, Yokosuka, Kanagawa 237-0061 Japan; 5 Submarine Resources Research Center, Research Institute for Marine Resources Utilization, Japan Agency for Marine-Earth Science and Technology (JAMSTEC), 2-15 Natsushima-cho, Yokosuka, Kanagawa 237-0061 Japan; 6 Department of Planetology, Kobe University, 1-1 Rokkodai-cho, Nada-ku, Kobe, Hyogo 657-8501 Japan; 7 Faculty of Science and Engineering, Waseda University, 3-4-1 Okubo, Shinjuku-ku, Tokyo, 169-8555 Japan

Correction to: *Scientific Reports*https://doi.org/10.1038/s41598-020-66835-8, published online 18 June 2020

The original version of this Article contained typographical errors in the Methods section.

As a result, under the subheading “Os and Re isotope analyses”,

“In addition to the samples from the study cores, we measured several uppermost sediment samples (0.00–0.02 mbsf) collected near core KR13-02 PC05 on cruises KR13-02 and MR14-E02 (Supplementary Table S3) to confirm that our Os isotopic data represented marine ^187^Os/^188^Os values. The ^187^Os/^188^Os ratios of these samples ranged from 0.954 ± 0.011 to 0.997 ± 0.012 (Supplementary Table S3).”

now reads:

“In addition to the samples from the study cores, we measured several uppermost sediment samples (0.00–0.02 mbsf) collected near core KR13-02 PC05 on cruises KR13-02 and MR14-E02 (Supplementary Table S2) to confirm that our Os isotopic data represented marine ^187^Os/^188^Os values. The ^187^Os/^188^Os ratios of these samples ranged from 0.954 ± 0.011 to 0.997 ± 0.012 (Supplementary Table S2).”

Furthermore, under the subheading “Marine ^187^Os/^188^Os curve since 40 Ma”,

“To complement the reference curve, we employed a long-term record of marine ^187^Os/^188^Os ratios obtained from a Fe–Mn crust in the Pacific^63^ refined by the thallium isotope record in the crust^16^ (Supplementary Fig. S1).”

now reads:

“To complement the reference curve, we employed a long-term record of marine ^187^Os/^188^Os ratios obtained from a Fe–Mn crust in the Pacific^63^ refined by the thallium isotope record in the crust^16^ (Supplementary Fig. S4).”

Additionally, in the Supplementary Information file originally published with this Article, Supplementary Tables S1–S3 were omitted.

These errors have now been corrected in the HTML and PDF versions of this Article and in the accompanying Supplementary Information.

