# Supplementary material for: Fish proliferation and rare-earth deposition by topographically induced upwelling at the late Eocene cooling event
Source: Sci Rep. 2020 Jun 18;10:9896. doi: 10.1038/s41598-020-66835-8 (PMC7303186; doi:10.1038/s41598-020-66835-8)
Supplement: Supplementary file 1 — Supplementary information. [file 41598_2020_66835_MOESM1_ESM.pdf]

Supplementary Information for

Fish proliferation and rare-earth deposition by topographically induced upwelling at the late Eocene cooling event

Junichiro Ohta, Kazutaka Yasukawa, Tatsuo Nozaki, Yutaro Takaya, Kazuhide Mimura, Koichiro Fujinaga, Kentaro Nakamura, Yoichi Usui, Jun-Ichi Kimura, Qing Chang, Yasuhiro Kato

Supplementary information includes:

- Supplementary Tables S1 to S3
- Supplementary Figs. S1 to S9

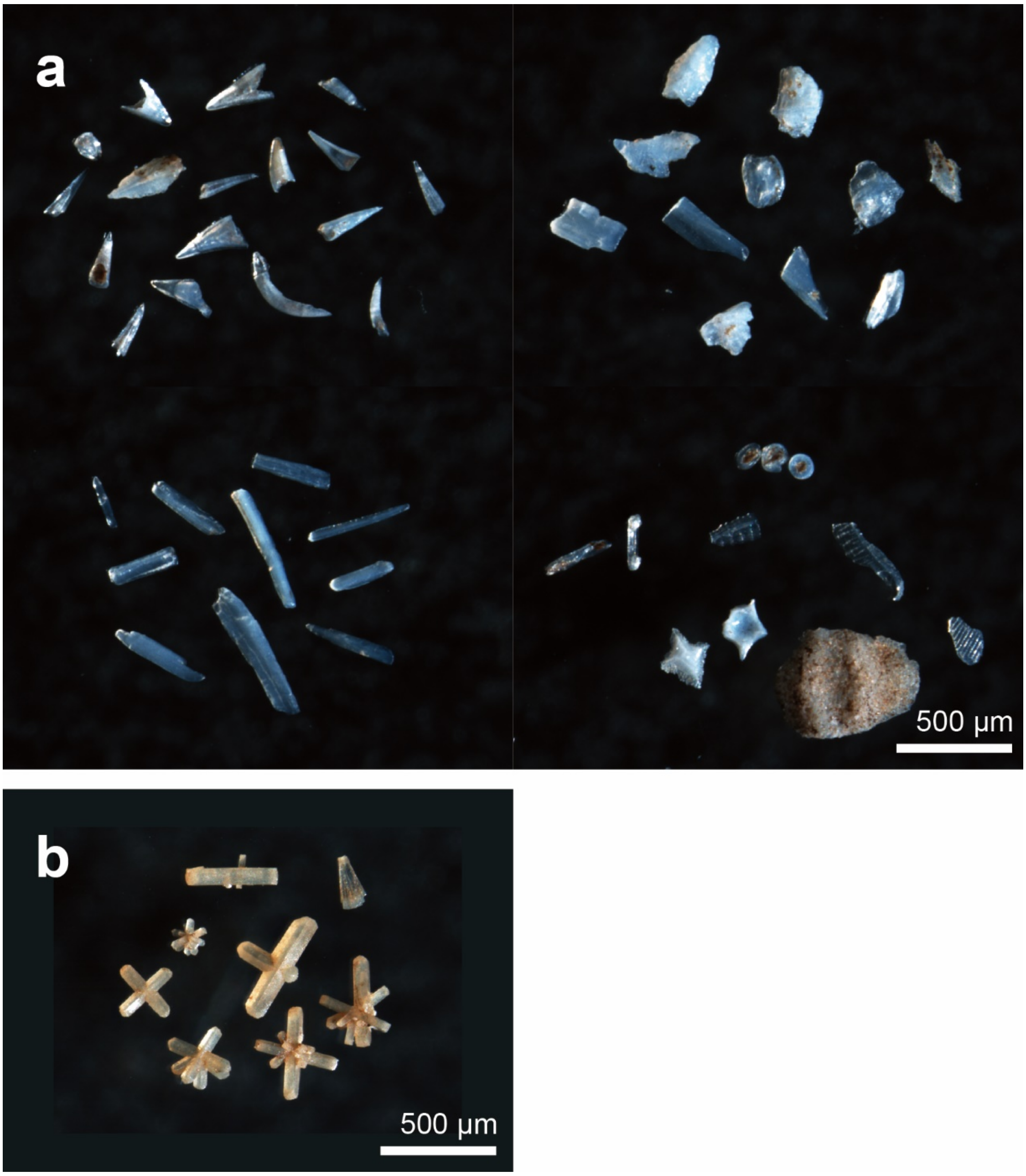

Fig. S1. Microphotographs of fish debris and phillipsite crystals. (a) fish debris, including teeth, denticles, and bone fragments, and (b) phillipsite crystals showing typical cruciform twin, separated from core KR13-02 PC05, Section 4, 72–74 cm taken under a stereomicroscope. All images were generated by focus stacking using Adobe Photoshop CS6.

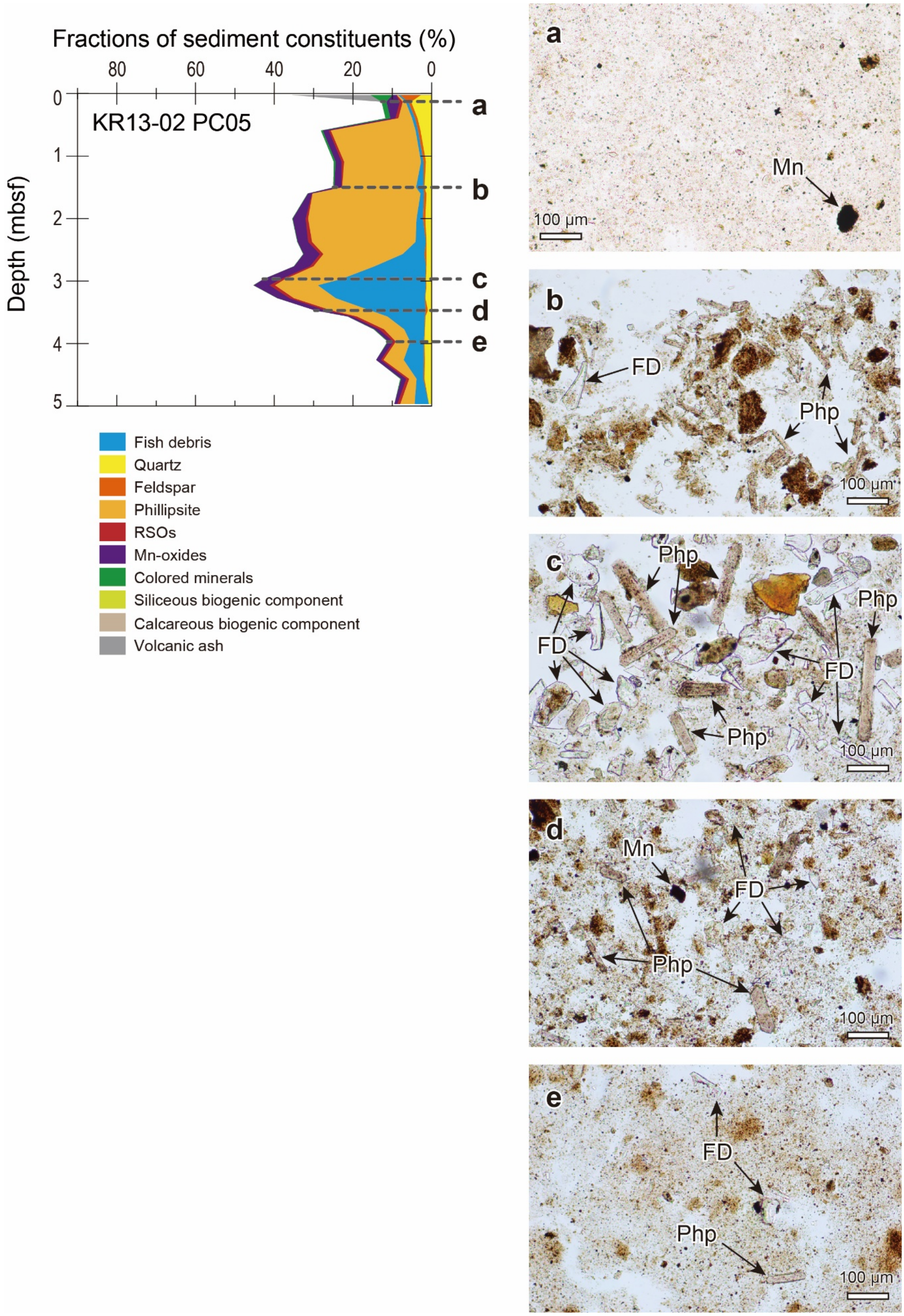

Fig. S2. Fractions of sediment constituents of KR13-02 PC05 with microphotographs of the representative horizons. (a) Section 1, 12–14 cm, 0.13 mbsf, (b) Section 3, 22–24 cm, 1.50 mbsf, (c) Section 4, 72–74 cm, 2.97 mbsf, (d) Section 5, 22–24 cm, 3.47 mbsf, and (e) Section 5, 72–74 cm, 3.97 mbsf. Abbreviations: FD = fish debris, Php = phillipsite, Mn = micro-manganese oxides. Images of (c) and (d) were generated by focus stacking using Adobe Photoshop CS6.

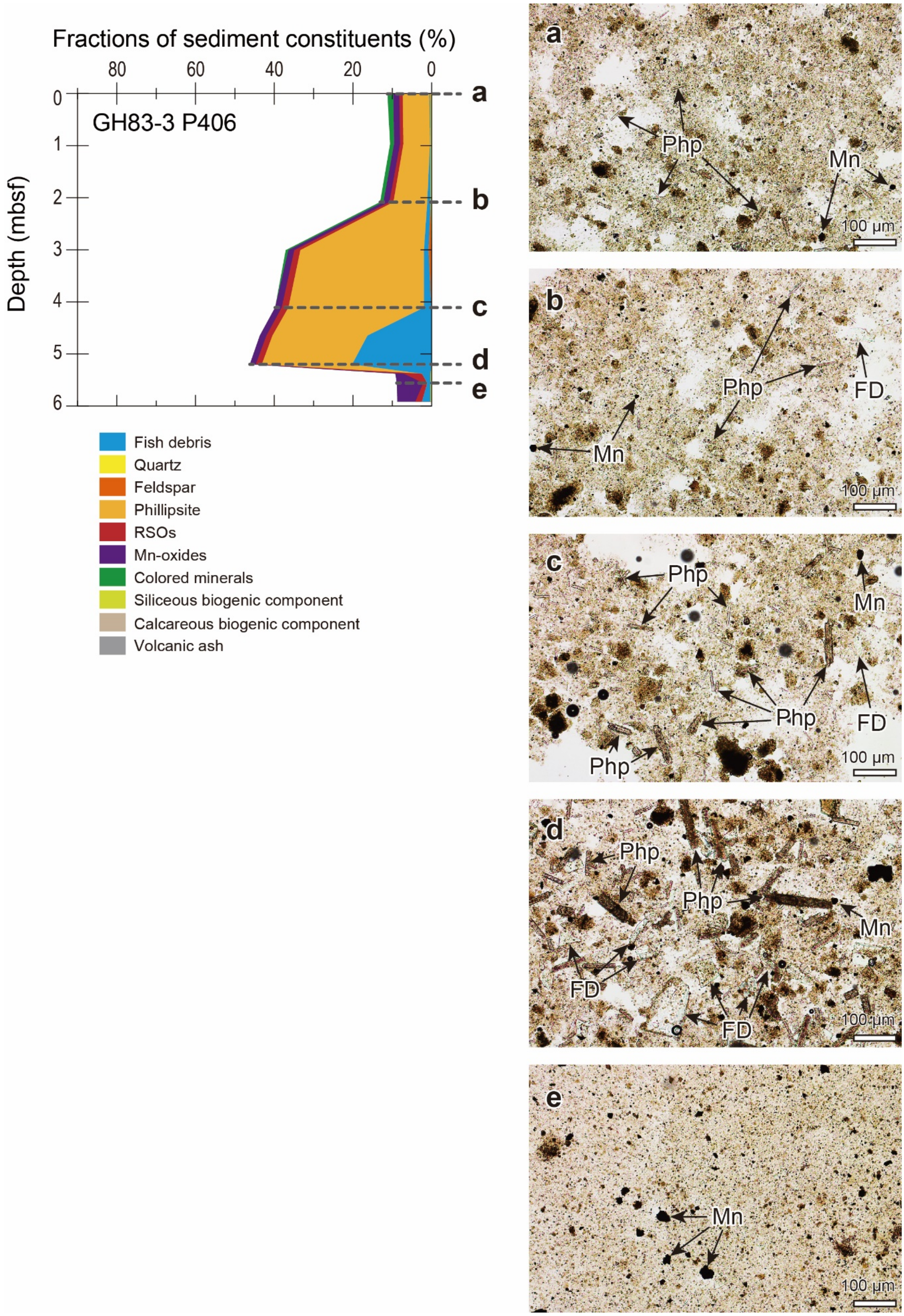

Fig. S3. Fractions of sediment constituents of GH83-3 P406 with microphotographs of the representative horizons. (a) Section I, 0–2 cm, 0.01 mbsf, (b) Section III, 52–54 cm, 2.08 mbsf, (c) Section V, 55–57 cm, 4.11 mbsf, (d) Section VI, 64–66 cm, 5.20 mbsf, and (e) Section VII, 0–2 cm, 5.56 mbsf. Abbreviations: FD = fish debris, Php = phillipsite, Mn = micro-manganese oxides.

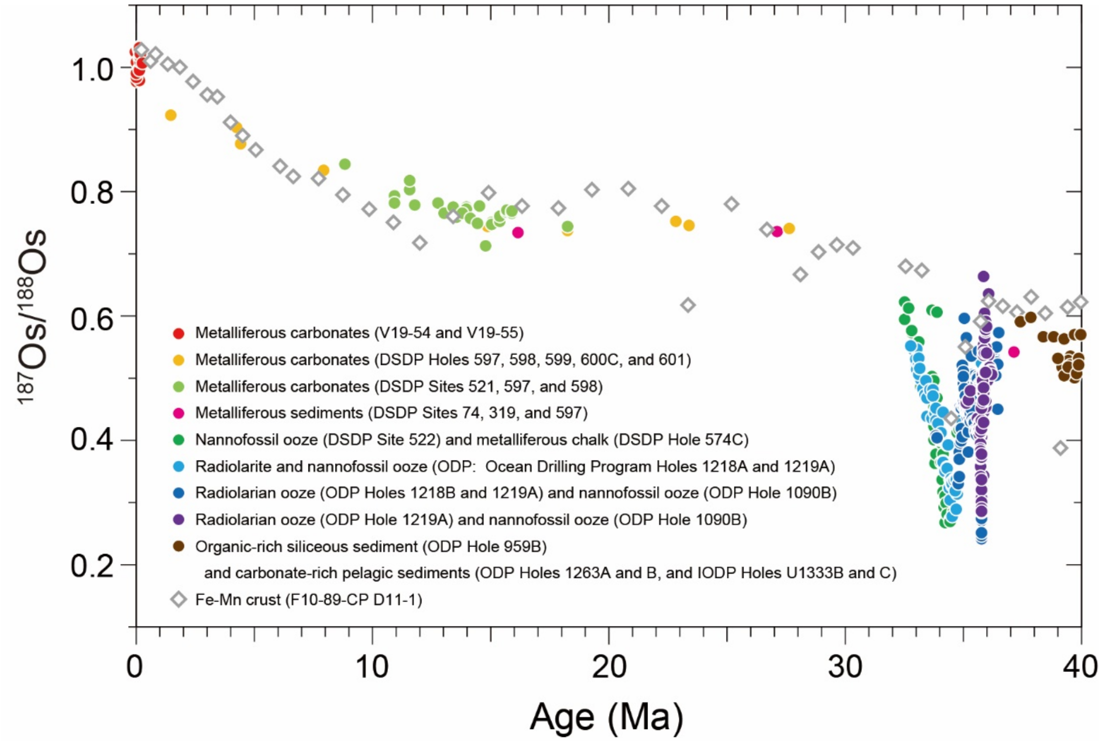

Fig. S4. The reference curve of marine $^{187}Os/^{188}Os$ compiled from previously reported $^{187}Os/^{188}Os$ data obtained from well-dated deep-sea sediments and Fe-Mn crust. Red circles are data from the sediment cores of metalliferous carbonates from the flanks of the East Pacific Rise (EPR; V19-54 and V19-55)[53]. Yellow circles indicate data from metalliferous carbonates from near the EPR (DSDP Holes 597, 598, 599, 600C, and 601)[54]. Light-green circles indicate data from metalliferous carbonates from near the EPR (DSDP Sites 597 and 598) and the Mid-Atlantic Ridge (DSDP Site 521)[55]. Pink circles indicate data from metalliferous sediments from near the EPR (DSDP Sites 74, 319, and 597)[56]. Green circles indicate data from nannofossil ooze from the South Atlantic (DSDP Site 522) and metalliferous chalk from the equatorial Pacific (DSDP Hole 574C)[57]. Light-blue circles indicate data from radiolarite and nannofossil ooze from the equatorial Pacific (ODP: Ocean Drilling Program Holes 1218A and 1219A)[58]. Blue circles indicate data from radiolarian ooze from the equatorial Pacific (ODP Holes 1218B and 1219A) and nannofossil ooze from the Southern Atlantic (ODP Hole 1090B)[59]. Purple circles indicate data from radiolarian ooze from the equatorial Pacific (ODP Hole 1219A) and nannofossil ooze from the Southern Atlantic (ODP Hole 1090B)[60]. Brown circles indicate data from organic-rich siliceous sediment from the equatorial Atlantic (ODP Hole 959B) as well as carbonate-rich pelagic sediments from the South Atlantic (ODP Holes 1263A and B) and from the equatorial Pacific (IODP: Integrated Ocean Drilling Program Holes U1333B and C)[61]. Grey open diamonds indicate data from Fe-Mn crust from the equatorial Pacific (F10-89-CP D11-1)[16].

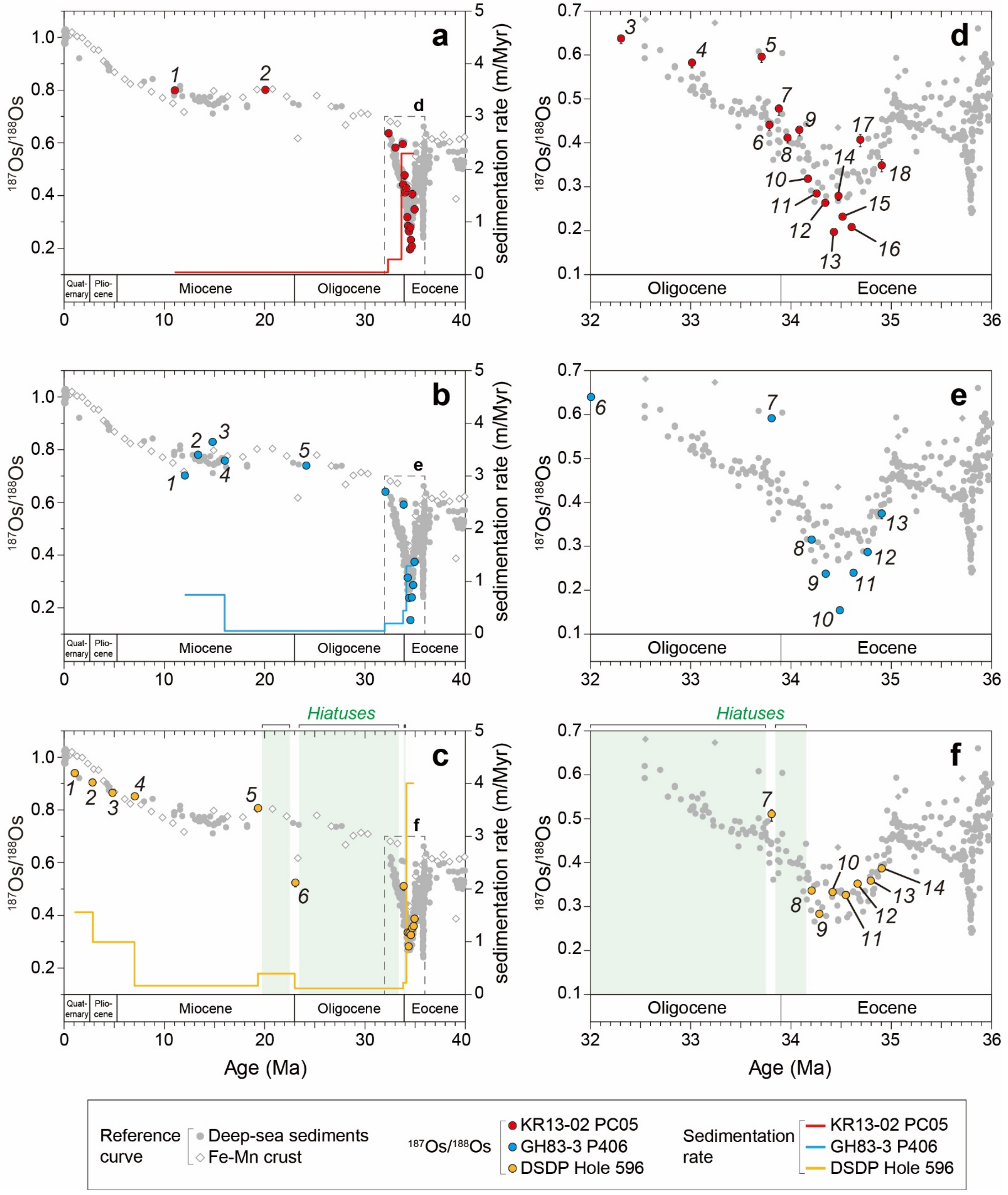

Fig. S5. Descriptive figure of our age assignment procedure with sample numbers. (a–c) The best fits of the $^{187}Os/^{188}Os$ data of core (a) KR13-02 PC05, (b) GH83-3 P406, and (c) DSDP Hole 596 with the reference curve of marine $^{187}Os/^{188}Os$. Sedimentation rates of these cores calculated from best-fit ages (see Methods) are also shown. (d–f) Enlarged views of panels a, b, and c, respectively. The green-shaded bands in (c) and (f) indicate probable hiatuses at 10.7 mbsf suggested by an abrupt change in lithology[18] and at 10.2 and 11.7 mbsf suggested by abrupt shifts in chemistry and mineralogy[24] in the core DSDP Hole 596.

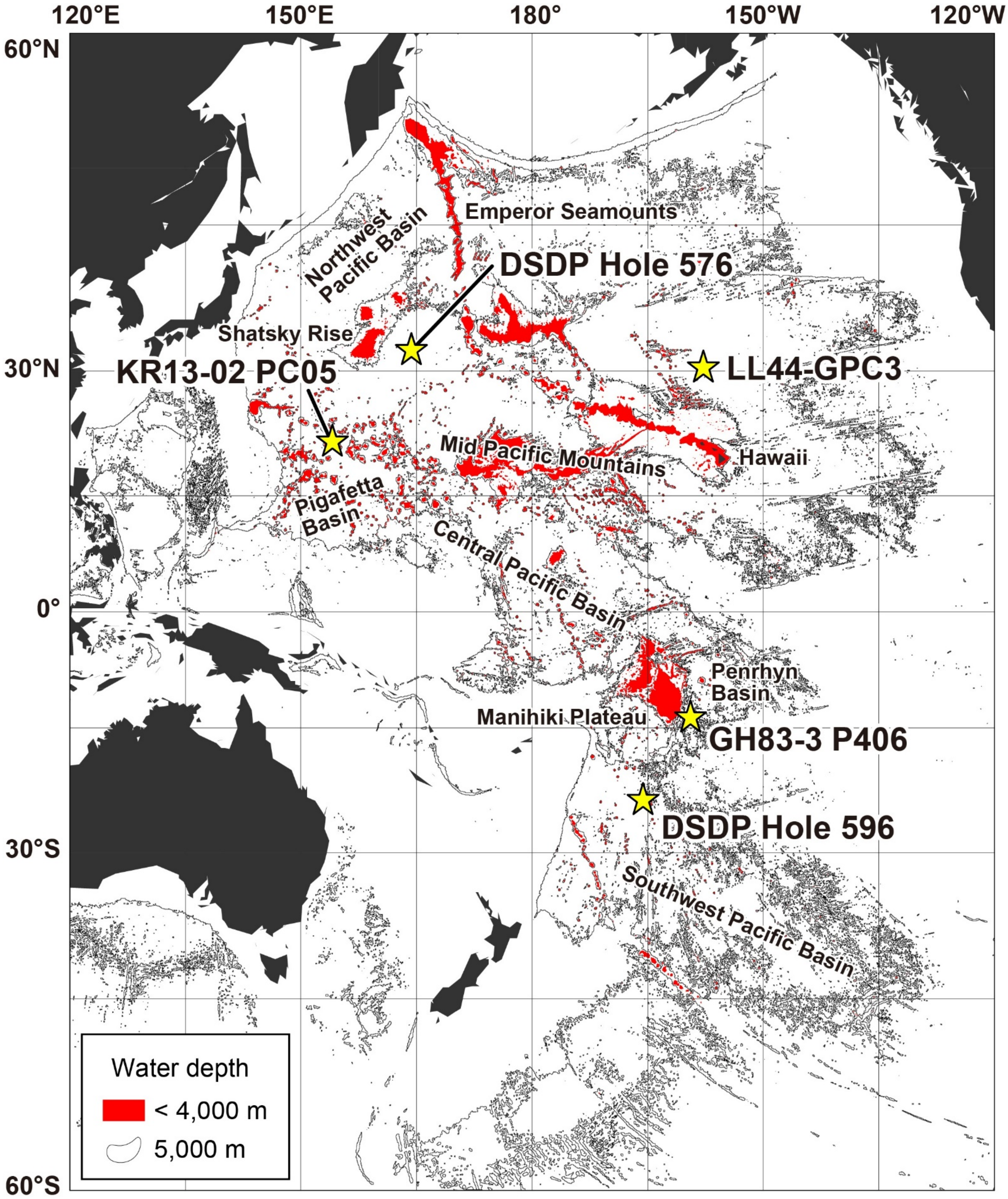

Fig. S6. Distribution of the topographic highs in the Pacific Ocean. Red areas indicate water depths shallower than 4,000 m distributed in basins greater than 5,000 m deep. Bathymetric data are from ETOPO1[68] (NOAA National Geophysical Data Center: 10.7289/V5C8276M; https://www.ngdc.noaa.gov/mgg/global/global.html). This map was created by using Generic Mapping Tools software, Version 4.5.18[69] (https://www.soest.hawaii.edu/gmt/).

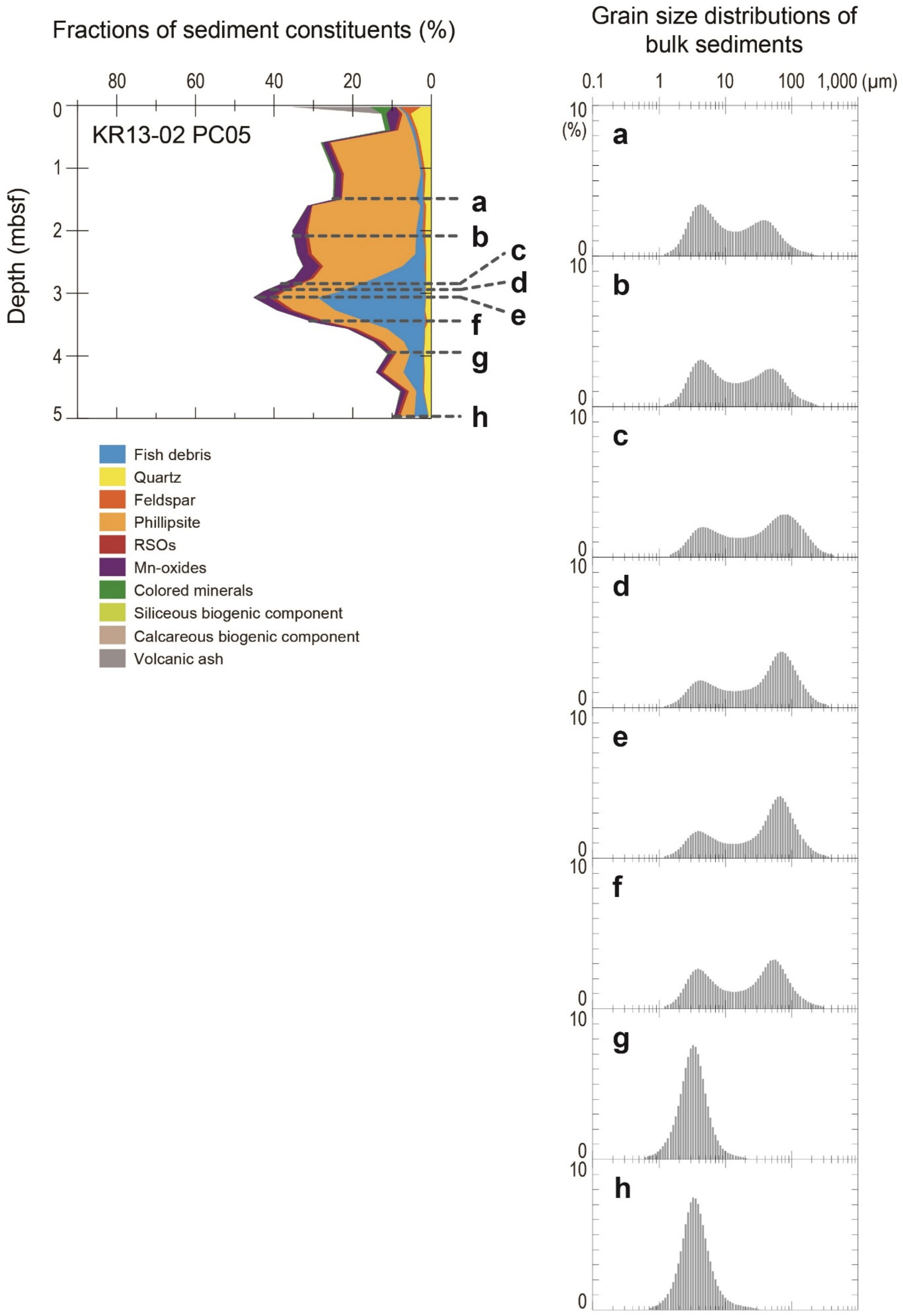

Fig. S7. Fractions of sediment constituents of KR13-02 PC05 with grain size distributions of bulk sediments. (a) Section 3, 22–24 cm, 1.50 mbsf, (b) Section 3, 82–84 cm, 2.10 mbsf, (c) Section 4, 62–64 cm, 2.87 mbsf, (d) Section 4, 72–74 cm, 2.97 mbsf, (e) Section 4, 82–84 cm, 3.07 mbsf, (f) Section 5, 22–24 cm, 3.47 mbsf, (g) Section 5, 72–74 cm, 3.97 mbsf, (h) Section 6, 72–74 cm, 4.97 mbsf. Panels a–h are modified from previously reported ones[13].

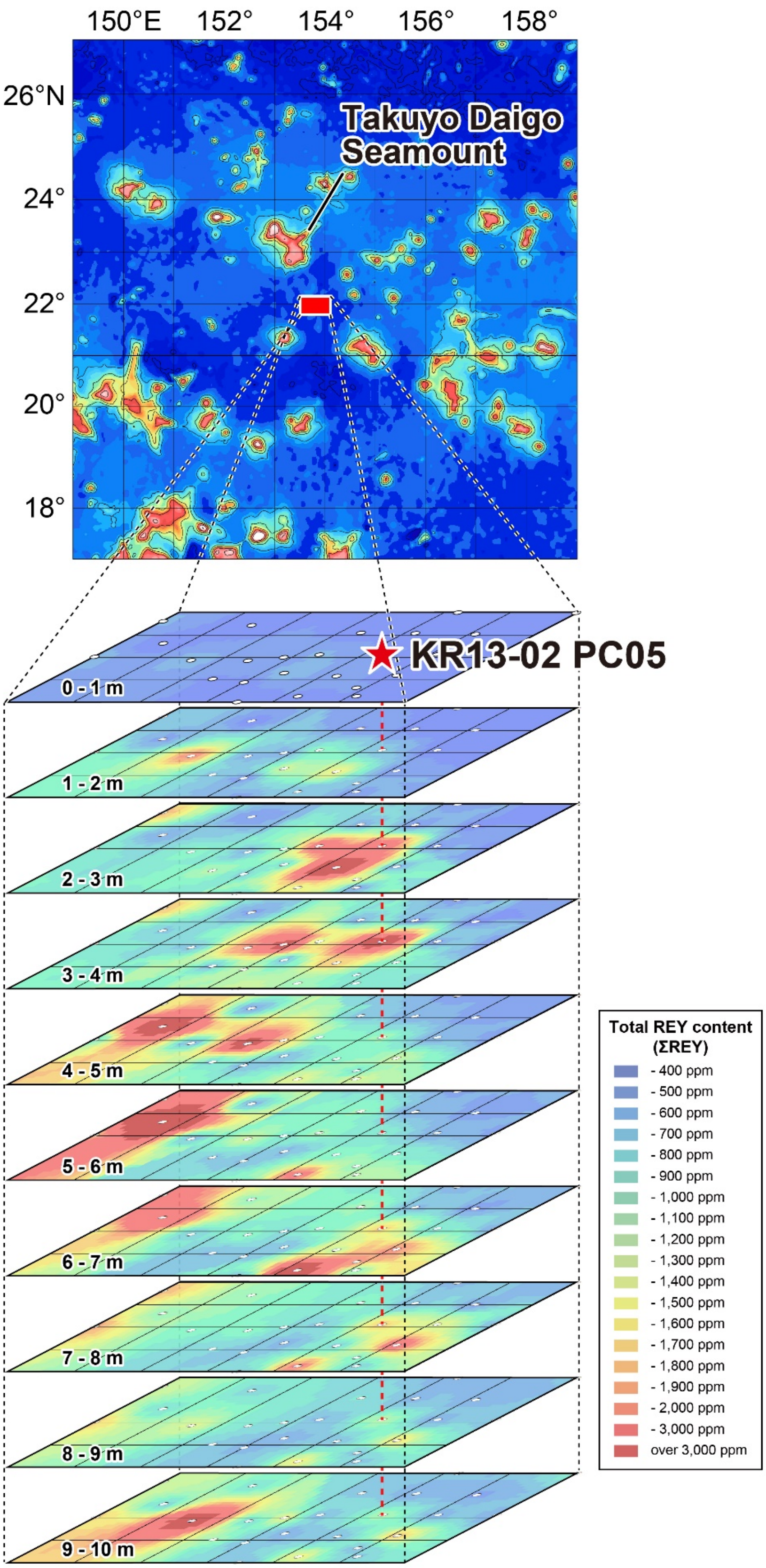

Fig. S8. Three-dimensional distribution of average total REY contents of deep-sea sediments for every meter below the seafloor within the area (~2,500 km^2) that holds promise for REY resource development south of the Takuyo Daigo Seamount. Colours correspond to the REY content: darker red areas have higher REY contents and thus hold promise as a resource for the elements. The red star indicates KR13-02 PC05, and tiny white circles represent other coring sites[9]. This figure is modified from previously reported one[9].

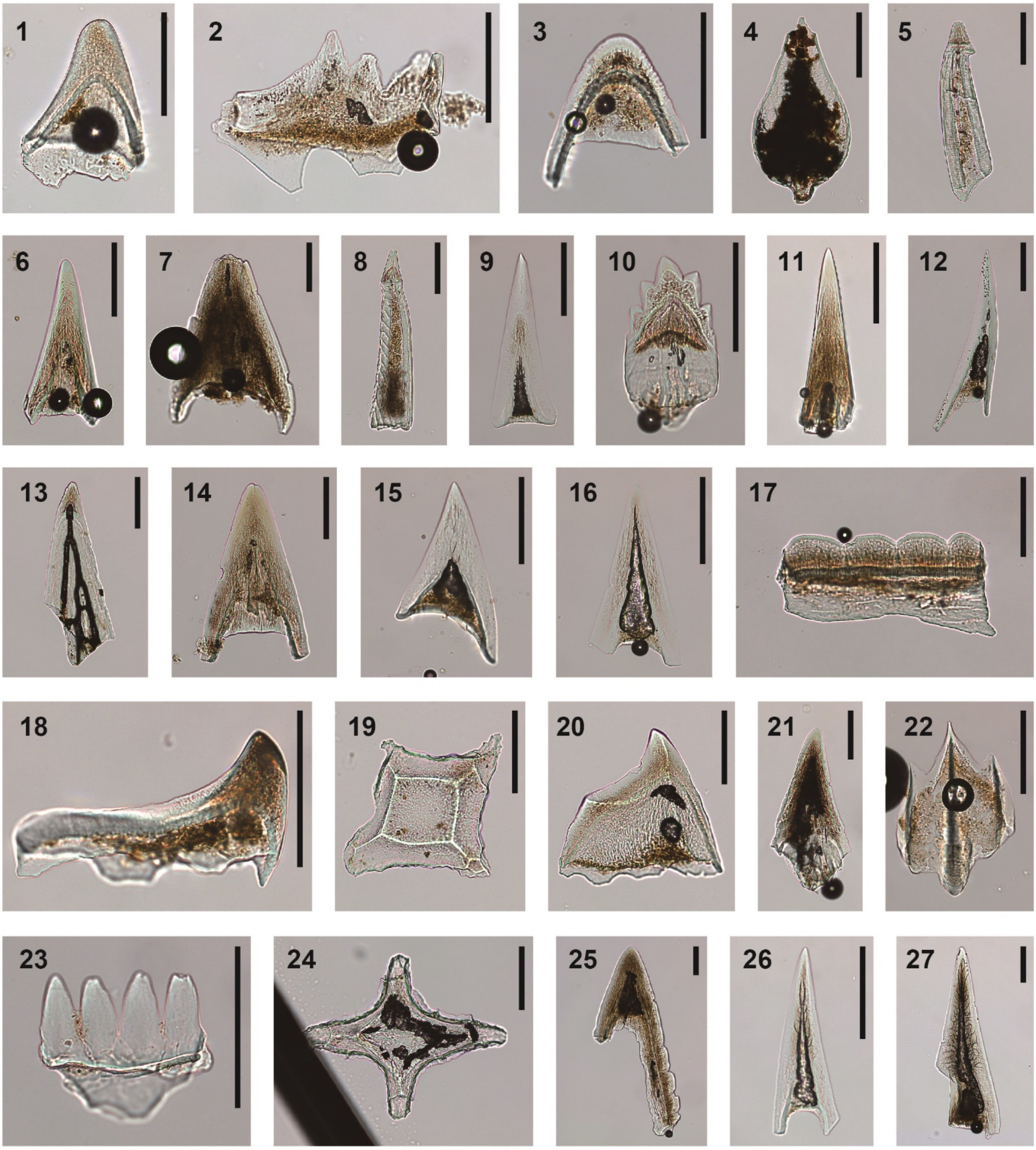

Fig. S9. Microphotographs of representative specimens of ichthyolith species identified in core KR13-02 PC05 taken under a polarizing microscope. (1) *Small triangle keeled edges*, (2) *asymmetrical peaks narrow depression*, (3) *wide crescent*, (4) *plain and lined lanceolate*, (5) *triangle with triangular projection*, (6) *triangle pointed margin ends*, (7) *triangle transverse line across*, (8) *narrow triangle cross-hachured*, (9) *triangle inline halfway*, (10) *small triangle crenate margin*, (11) *pointed triangle short inline*, (12) *triangle short wing*, (13) *triangle one canal above*, (14) *wide triangle straight inbase*, (15) *curved triangle pointed inline*, (16) *flexed triangle shallow inbase*, (17) *rectangular irregularly saw-toothed*, (18) *asymmetrical peak wide depression*, (19) *polygonal cavity*, (20) *asymmetrical peak depression*, (21) *triangle with canals*, (22) *short side peaks differentiated margin*, (23) *long saw-toothed*, (24) *polygonal cavity long rays*, (25) *triangle crenulate*, (26) *flexed triangle shallow inbase ≥ 120*, (27) *flexed triangle 115-118*. Scale bars = 100 µm.
